# Collateral beauty: left main stem atresia

**DOI:** 10.1093/ehjcr/ytae241

**Published:** 2024-05-08

**Authors:** Sara Ranchordás, Adriana Vazão, Francisco Soares, Marta Marques

**Affiliations:** Cardiac Surgery, Hospital Santa Cruz, ULSLO, Av. Professor Reinaldo dos Santos, 2790-134 Carnaxide, Portugal; Cardiology, Centro Hospitalar de Leiria, Leiria, Portugal; Cardiology, Centro Hospitalar de Leiria, Leiria, Portugal; Cardiac Surgery, Hospital Santa Cruz, ULSLO, Av. Professor Reinaldo dos Santos, 2790-134 Carnaxide, Portugal

## Case description

**Figure 1:**
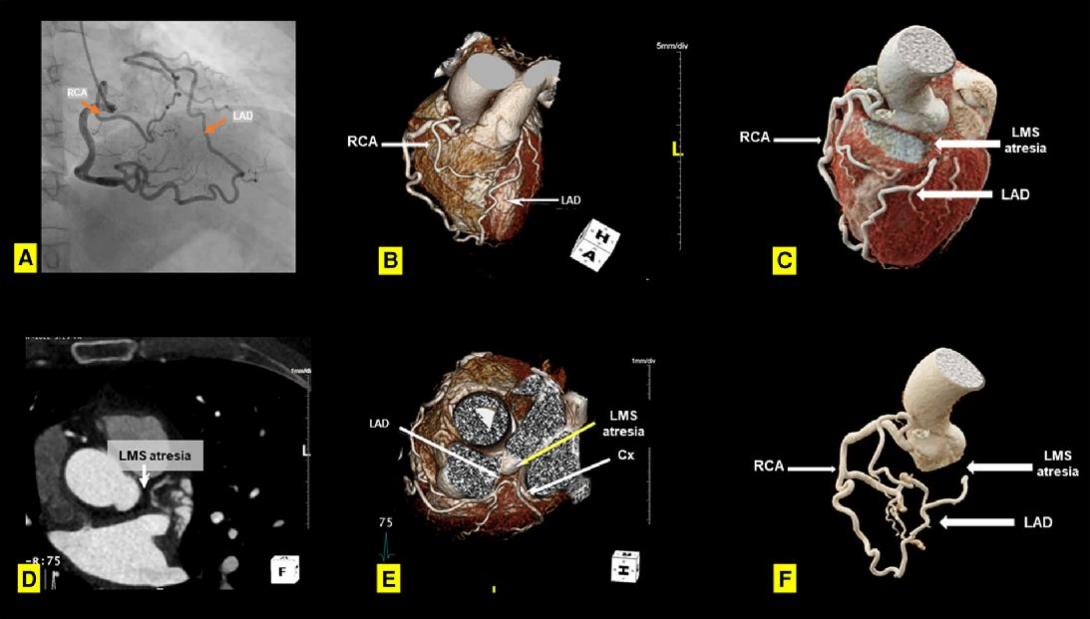
(*A*) Invasive coronary angiography. Right coronary catheterization showing left main stem atresia with collaterals from the right coronary artery supplying retrogradely the left coronary circulation. (*B–F*) Computed tomography coronary angiography. (*B* and *C*) 3D reconstruction showing collaterals between left anterior descending and right coronary artery and left main stem atresia. (*D*) 2D axial plane showing left main stem atresia. (*E*) 3D reconstruction showing left main stem atresia. (*F*) 3D reconstruction of the coronary tree showing collaterals between left anterior descending artery and right coronary artery and left main stem atresia. Cx, circumflex artery; LAD, left anterior descending artery; LMS, left main stem; RCA, right coronary artery.

A 53-year-old man with arterial hypertension and diabetes underwent exams for an elective cholecystectomy. The patient reported only nonspecific complaints of body numbness. He had undergone amputation of both legs due to electrocution over 20 years before. He underwent Holter monitoring, which showed dynamic ventricular repolarization changes. Invasive coronary angiography (*Figure 1A*) followed by computed tomography coronary angiography (*Figure 1B–F*) showed left main stem (LMS) atresia with collaterals from the right coronary artery (RCA) supplying retrogradely the left coronary circulation. The echocardiogram showed good biventricular function, with no regional wall motion abnormalities. Cardiac magnetic resonance myocardial perfusion imaging showed no ischaemia. During the 18-month follow-up period, the patient remained asymptomatic for ischaemic events, and therefore, no intervention was deemed necessary. He continued his usual medication, including clopidogrel.

Left main stem atresia is an exceedingly rare coronary artery anomaly characterized by the absence of the LMS, resulting in the proximal blind ending of the left anterior descending and circumflex arteries, which are supplied by collateral circulation from the RCA, unlike the differential diagnosis single coronary artery malformation. When there is a single coronary blood supply, direction is antegrade, flowing from the single coronary to its branches.^1,2^

The diagnosis is generally made early in life, if there are symptoms, such as angina, syncope, or sudden cardiac death. However, in rare cases, it is only diagnosed in adulthood, frequently as an incidental finding, due to the development of sufficient collateral circulation.^1^

When symptomatic, coronary artery bypass surgery or surgical coronary repair is recommended. However, in asymptomatic adult patients with no evidence of inducible myocardial ischaemia, conservative management with optimal medical therapy is reasonable.^3^

## Data Availability

The data underlying this article are available in the article.

## Notes

**Consent:** Informed patient consent was obtained from the patient.

**Funding:** None declared.
